# The value of pupillary diameter in evaluating pain perception after awakening in patients undergoing general anesthesia during orthopedic surgery

**DOI:** 10.1186/s12871-024-02428-6

**Published:** 2024-02-09

**Authors:** Huang Huang, Yinuo Qiu, Guoxin Gu, Xiangyang Mei, Liwei Pang, Chuangxin Zhang, Mingzi Ran, Mengmeng Li

**Affiliations:** 1 Center for Rehabilitation Medicine, Department of Anesthesiology, Zhejiang Provincial People’s Hospital, Affiliated People’s Hospital, Hangzhou Medical College, Hangzhou, 310014 Zhejiang China; 2 Department of Anesthesiology, the Fourth Medical Center of Chinese PLA General Hospital, Beijing, 100048 China

**Keywords:** Noxious stimulation, Pupillary diameter, Pupillary reflex dilation, Pupillary light reflex

## Abstract

**Background:**

The pupillary response to tetanic electrical stimulation reflects the balance between nociceptive stimulation and analgesia. Although pupillary pain index (PPI) was utilized to predict postoperative pain, it depended on tetanic stimulation and was complex. We aim to describe the potential relationship between PD in the presence of surgical stimulation and pain levels after awakening.

**Methods:**

According to the Verbal Rating Scale (VRS) score after extubation, the patients were divided into painless group (VRS = 0) and pain group (VRS ≥ 1). Pupillary diameter (PD) and pupillary light reflex velocity (PLRV) were compared between two groups when patients entered the operating room (T_1_), before incision (T_2_), 10 s after incision (T_3_), 30 s after incision (T_4_), 1 h after incision (T_5_), at the end of surgery (T_6_), shortly after extubation (T_7_), and when patients expressed pain clearly (T_8_). The magnitude of PD change (ΔPD) compared to the baseline value after anesthesia induction (T_2_) was calculated. The correlations between pupillary parameters and pain after awakening were calculated.

**Results:**

Patients with VRS ≥ 1 had greater PD than painless patients at T_3-7_ (*P* = 0.04, 0.04, 0.003, <0.001, <0.001), and it was positively correlated with VRS score after awakening at T_4-7_ (*r* = 0.188, 0.217, 0.684, 0.721). The ability of T_6_ΔPD to predict VRS ≥ 1 was strong [threshold: 20.53%, area under the curve (AUC): 0.93, 95% confidence interval (CI): 0.89–0.97 ].

**Conclusion:**

Our study indicates that PD is a useful index to direct the individualized analgesics used during operation, to better avoid the occurrence of pain during the postoperative emergence period.

**Trial registration:**

This study was registered with the Chinese Clinical Trial Registry (registration number: ChiCTR2000040908, registration date: 15/12/2020).

## Introduction

Rapid control of acute pain after extubation in patients undergoing general anesthesia is the key to postoperative pain management influencing overall satisfaction with postoperative analgesia [1]. However, due to residual anesthetics, the patient is not fully awake after surgery, and cannot accurately express pain experience, which makes precise pain management a challenge [2]. Previous studies have shown that pupillary monitoring is a method for assessment of the degree of nociception in perioperative patients, in addition to blood pressure and heart rate [3]. Due to the short dilation latency and obvious amplitude, evaluating nociception in patients undergoing general anesthesia is more advantageous [4].

Accurate instruments for pupillary monitoring have been upgraded in recent years, mainly including infrared and electronic pupillary instruments promoting extensive studies on the perioperative management of the pupillary [5–8]. Previous studies predicted the target concentration of opioids or the degree of pain after awakening using a standard tetanic electrical stimulation to monitor the pupillary response [9]. To assess the pupillary reflex dilation (PRD), pupillary pain index (PPI) was used to evaluate the response of a continuously increasing electric stimulus discharge from 10 to 60 mA. The response was then classified on a scale from 1 to 9, positively correlated with nociception [9]. Nociceptive stimulation inhibits the Edinger-Westphal nucleus to dilate the pupillary, and the degree of dilation is positively correlated with the intensity of the nociceptive stimulation. Opioid drugs inhibit PRD after nociceptive stimulation by blocking nerve conduction between brainstem inhibitory neurons and the Edinger-Westphal nucleus [8]. Therefore, although the intensity of skin incision stimulation and the concentration of opioids cannot be accurately quantified intraoperatively, the dynamic changes in pupillary diameter (PD) can reflect the balance of noxious stimulation and analgesia. Theoretically, there is a threshold value for PD. When it exceeds this threshold value, it indicates that the patient may feel pain after waking. In our study, PD was regarded as the result of the interaction between surgical stimulation and analgesia, the variations of PD at different time points during surgery were assessed, and it was attempted to indicate whether it could be used to predict patients’ pain perception after awakening.

## Methods

### Ethics approval

This study was approved by the Ethics Committee of the Fourth Medical Center of Chinese PLA General Hospital (Approval number: 2020KY041-HS001) and registered in the China Clinical Trials Registry (registration number: ChiCTR2000040908, registration date: 15/12/2020) before being conducted. All patients signed the written informed consent form. Inclusion criteria were patients aged 18–65 years old with clear awareness and good communication and scheduled for orthopedic surgery under general anesthesia in the Fourth Medical Center of Chinese PLA General Hospital (Beijing, China) between February 2021 and February 2022, including 65 patients undergoing knee joint replacement, 56 patients receiving hip joint replacement, and 59 patients undergoing periacetabular osteotomy. Exclusion criteria were patients with iris disease, cataracts and other eye diseases or history of trauma, history of consumption of cardiovascular active drugs, history of consumption of anticholinesterase/anticholinergic drugs in the perioperative period, and unequal pupil size before and after extubation.

### Anesthetic management during the study period

After the patient entered the operating room, venous access was established. Electrocardiogram, invasive blood pressure, oxygen saturation, and bispectral index (BIS) were monitored. Midazolam (0.02 mg kg^-1^), sufentanil (0.2 µg kg^-1^), propofol (1–2 mg kg^-1^), and rocuronium (0.6 mg kg^-1^) were used for induction of anesthesia. Then, tracheal intubation was performed after 3 to 5 min to control breathing by mechanical ventilation. Tetanic stimulation was surgical stimulation including skin, muscles, and bones. Before skin incision, the dosage of anesthetics should be adjusted according to the patient’s circulation to maintain the hemodynamic fluctuation within ± 20% of the preoperative baseline value, and the BIS should be between 40 and 60. After the surgery, the anesthetic drug infusion was stopped, and the tracheal tube was removed after the patient was awake and spontaneous breathing resumed.

### Data Collection

A portable electronic pupillometer (TK500A; Shaanxi Public Intelligence Technology Co., Ltd., Shaanxi, China; License No. 20,202,160,003) was used to measure patients’ PD and pupillary light reflex velocity (PLRV). During the examination, the brightness of the background light source was 750 lx, and the independent measurement mode was adopted. The patient’s right eye was closed and the left eyelid was opened to expose the complete pupil. The front end of the instrument collection area was completely attached to the upper part of the left eye so that the eye was placed in the center of the measurement window. When the top surface was flush with the front plane of the acquisition window, the measurement key was pressed to obtain PD and PLRV.

The preoperative pain perception was evaluated by the verbal rating scale (VRS), in which VRS of 0 point indicated no pain, VRS of 1 point represented mild pain, VRS of 2 points indicated moderate pain, VRS of 3 points represented severe pain. When patients entered the operating room (T_1_), before incision (T_2_), 10 s after incision (T_3_), 30 s after incision (T_4_), 1 h after incision (T_5_), at the end of the surgery (T_6_), immediately after intubation (T_7_), and when patients expressed pain clearly (T_8_), PD and PLRV were evaluated. The change in PD was calculated based on the basic value after anesthesia induction to reflect the impact of surgical stimulation on PD.

$${T_n}\Delta PD = ({T_n}PD - {T_2}PD)/{T_2}PD$$

After patients woke up and extubation, pain assessment was completed within 10 min. Patients with VRS equal to 0 point were assigned to the painless group. Patients with VRS ≥ 1 point were assigned to the pain group, and patients with VRS ≥ 2 points were treated with sufentanil (5 µg) for analgesic rescue [1].

### Statistical analysis

According to the results of pre-experiment analysis of 30 patients, the minimum area under the receiver operating characteristic (ROC) curve was set to 0.5. The confidence level was 1-α = 0.95, and the power was 1-β = 0.8. The minimum sample size of **160** subjects was confirmed by PASS 15.0 software. Considering a 10% dropout rate, 180 cases were enrolled in this study. SPSS 26.0 software (IBM, Armonk, NY, USA) was used to analyze and process the data. Normal distribution of continuous data was evaluated using the Shapiro–Wilk test. Normally distributed variables were reported as mean (standard deviation [SD]), and Repeated analysis of measurement variance (ANOVA) was used to make the comparison between the pain group and the painless group. The enumeration data were expressed as the number of cases, and the comparison was performed using the Chi-square test. Non-normally distributed values were reported as median (25th to 75th percentiles), and the Mann–Whitney *U* test was used to analyze ΔPD between two groups. Spearman test was used to analyze the correlation between PD at different time points and VRS after awakening. ROC curves were plotted by MedCalc software (MedCalc Software Ltd., Ostend, Belgium) to analyze the predictive performance of each index. *P*-value < 0.05 was considered statistically significant.

## Results

### General data

**Fig. 1:**
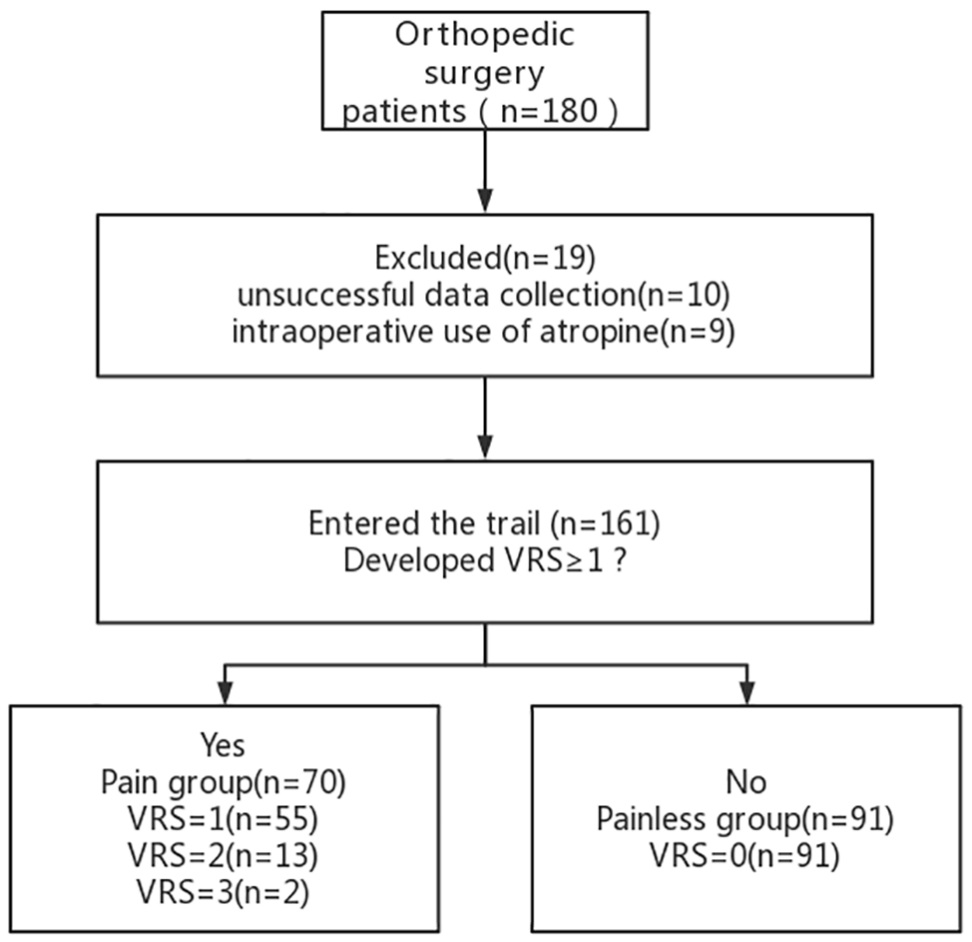
Study flowchart

This study enrolled 180 subjects, of whom 10 cases were excluded due to unsuccessful data collection, 9 patients were excluded because of intraoperative use of atropine, and 161 patients were ultimately enrolled. At T_8_, 91 cases with VRS of 0 point were selected in the painless group, and 70 cases with VRS ≥ 1 point were selected in the pain group, including 55 cases with mild pain, 13 cases with moderate pain, and 2 cases with severe pain. Only 1 case suffered from moderate pain when leaving the room (Fig. 1).

The general data were compared between the painless group and the pain group, and it was found that there was no significant difference in the general data, operation time, and intraoperative medication between the two groups (Table 1).

**Table 1:** Demographic data of two groups

Item	Painless group (*n* = 91)	Pain group (*n* = 70)	*P*
Age (years old)	48.78 ± 13.38	44.69 ± 14.58	0.066
Gender (*n*, male/female)	40/51	43/37	0.201
BMI (kg/m^2^)	25.02 ± 3.54	25.53 ± 3.57	0.373
ASA (*n*, I/II/III)	35/51/5	28/39/3	0.932
VRS before surgery (*n*, 0/1/2)	31/57/3	18/47/5	0.331
Type of surgery (*n*, knee/hip/osteotomy)	33/26/32	20/24/26	0.557
Operation time (min)	110.93 ± 21.61	119.57 ± 39.62	0.103
Sufentanil (g)	23.68 ± 6.69	25.39 ± 6.67	0.109
Remifentanil (mg)	1.03 ± 0.39	1.16 ± 0.55	0.071
Propofol (mg)	278.57 ± 112.04	328.14 ± 111.52	0.105
Sevoflurane (ml)	23.25 ± 9.57	25.87 ± 10.99	0.109

BMI: body mass index, ASA American Society of anesthesiologists physical status, VRS: verbal rating scale

### Comparison of PD and ΔPD between the painless group and the pain group

PD in the pain group was higher than that in the painless group at T_3-7_(*P* = 0.04, 0.04, 0.003, *P*<0.001, <0.001) (Table 2). ΔPD in the pain group was higher than that in the painless group at T_3-7_(*P*<0.001) (Table 3). The change in PD initially decreased, and then, gradually increased. Compared with T_1_, PD decreased at T_2-8_ (*P* < 0.001). Compared with T_2_, PD increased at T_3-8_ (*P* < 0.001).

**Table 2:** PD of two groups at T_1 ~ 8_

		T_1_	T_2_	T_3_	T_4_	T_5_	T_6_	T_7_	T_8_
PD (mm)	Painless group	3.11 ± 0.53	1.71 ± 0.19	1.77 ± 0.21	1.87 ± 0.23	1.80 ± 0.23	1.85 ± 0.21	1.95 ± 0.23	2.41 ± 0.34
	Pain group	3.01 ± 0.47	1.70 ± 0.22	1.85 ± 0.30	1.95 ± 0.26	1.92 ± 0.27	2.25 ± 0.24	2.50 ± 0.33	2.49 ± 0.37
	*P*	0.202	0.899	0.040	0.035	0.003	0.000	0.000	0.132

*PD: Pupillary diameter. T*_*1*_: *when patients entered the operating room, T*_*2*_: *before incision, T*_*3*_: *10 s after incision, T*_*4*_: *30 s after incision, T*_*5*_: *1 h after incision, T*_*6*_: *at the end of surgery, T*_*7*_: *shortly after extubation, T*_*8*_: *when patients expressed pain clearly*

**Table 3:** ΔPD of two groups at T_1 ~ 8_

		T_1_	T_3_	T_4_	T_5_	T_6_	T_7_	T_8_
ΔPD (%)	Painless group	83.33(61.11,106.25)	0(0,7.14)	6.25(0,15)	0(0,6.67)	6.25(4.55,12.5)	12.5(5.88,22.22)	41.18(23.53,56.25)
	Pain group	73.68(52.21,105.56)	0(0,14.29)	11.11(4.76,23.56)	13.39(5.48,21.43)	29.41(21.93,44.44)	48.53(33.33,61.11)	46.76(33.33,62.95)
	*P*	0.323	0.013	0.017	0.000	0.000	0.000	0.147

*PD: Pupillary diameter. T*_*1*_: *when patients entered the operating room, T*_*3*_: *10 s after incision, T*_*4*_: *30 s after incision, T*_*5*_: *1 h after incision, T*_*6*_: *at the end of surgery, T*_*7*_: *shortly after extubation, T*_*8*_: *when patients expressed pain clearly*

### Comparison of perioperative PLRV between the painless group and the pain group

There was no significant difference in PLRV between the two groups. PLRV showed an overall downward trend, followed by an upward trend over time. Compared with T_1_, PLRV decreased at T_2-8_ (*P* < 0.001). Compared with T_2_, PLRV increased at T_7-8_ (*P* < 0.001) (Table 4).

**Table 4:** PLRV of two groups at T_1 ~ 8_

		T_1_	T_2_	T_3_	T_4_	T_5_	T_6_	T_7_	T_8_
PLRV (mm/s)	Painless group	1.37 ± 0.66	0.54 ± 0.27	0.58 ± 0.31	0.60 ± 0.30	0.59 ± 0.23	0.58 ± 0.29	0.88 ± 0.59	1.10 ± 0.48
	Pain group	1.51 ± 0.71	0.61 ± 0.22	0.63 ± 0.23	0.64 ± 0.31	0.62 ± 0.18	0.65 ± 0.31	0.92 ± 0.65	1.06 ± 0.56
	*P*	0.183	0.083	0.280	0.298	0.310	0.197	0.672	0.586

*PLRV: Pupillary light reflex velocity. T*_*1*_: *when patients entered the operating room, T*_*2*_: *before incision, T*_*3*_: *10 s after incision, T*_*4*_: *30 s after incision, T*_*5*_: *1 h after incision, T*_*6*_: *at the end of surgery, T*_*7*_: *shortly after extubation, T*_*8*_: *when patients expressed pain clearly*

### Relationship between PD and VRS at each time point in the perioperative period

**Fig. 2:**
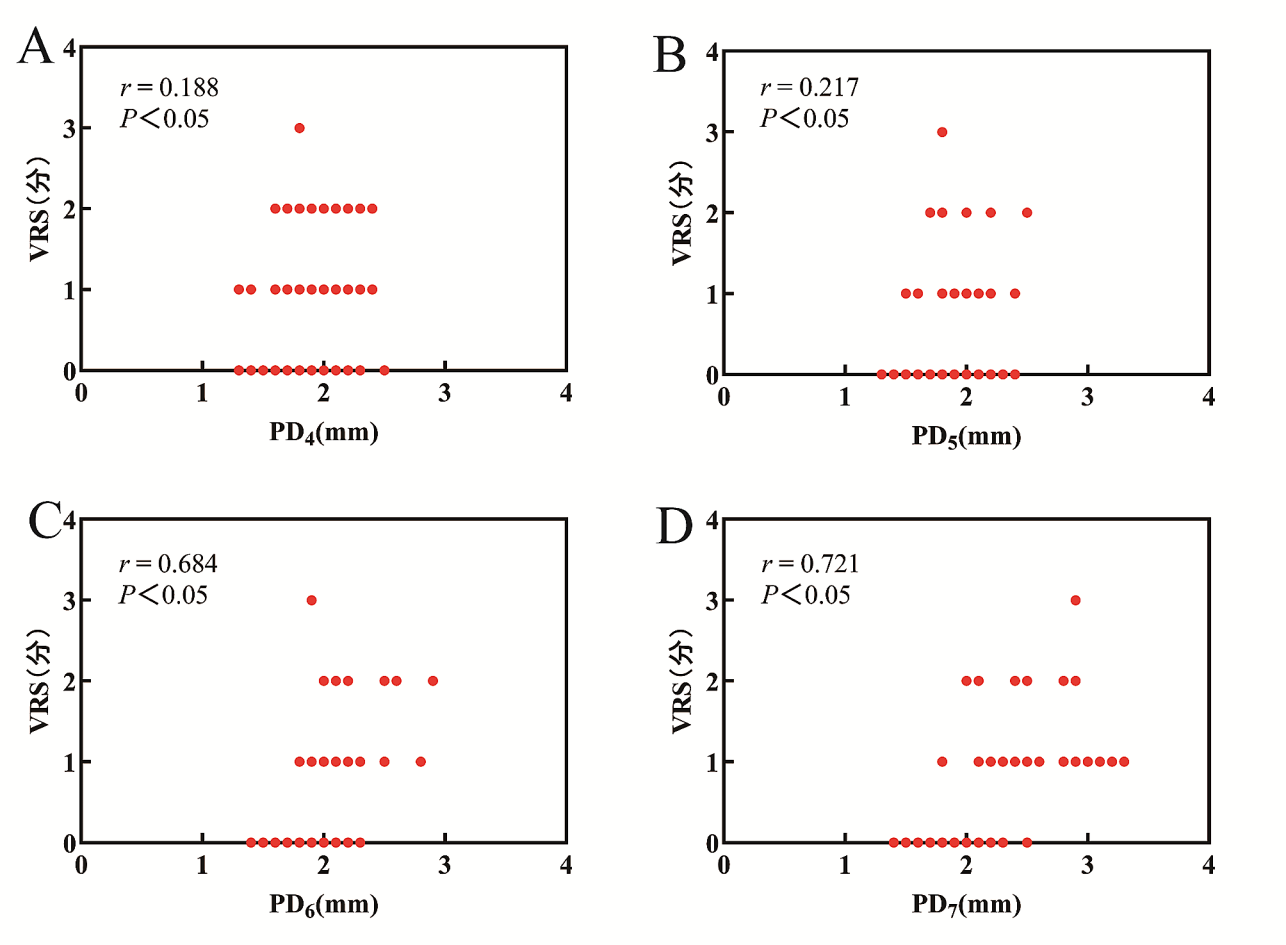
Graph showing the correlation between VRS and PD. ** (A)** Relationship between PD_4_ and VRS. **(B)** Relationship between PD_5_ and VRS. **(C)** Relationship between PD_6_ and VRS. **(D)** Relationship between PD_7_ and VRS. With the progress of surgery, *r*, correlation coefficient gradually increases. VRS: verbal rating scale. T_4_: 30 s after incision, T_5_: 1 h after incision, T_6_: at the end of surgery, T_7_: shortly after extubation

There was no correlation between preoperative PD and preoperative VRS score, while a correlation was found between PD and VRS score after awakening at T_4-7_ (*r* = 0.188, 0.217, 0.684, 0.721, *P* = 0.017, 0.006, <0.0005, <0.0005). However, there was no correlation between PD at T_3_ and T_8_ and VRS score after awakening (Fig. 2).

### The accuracy of each index in predicting pain after awakening

**Fig. 3:**
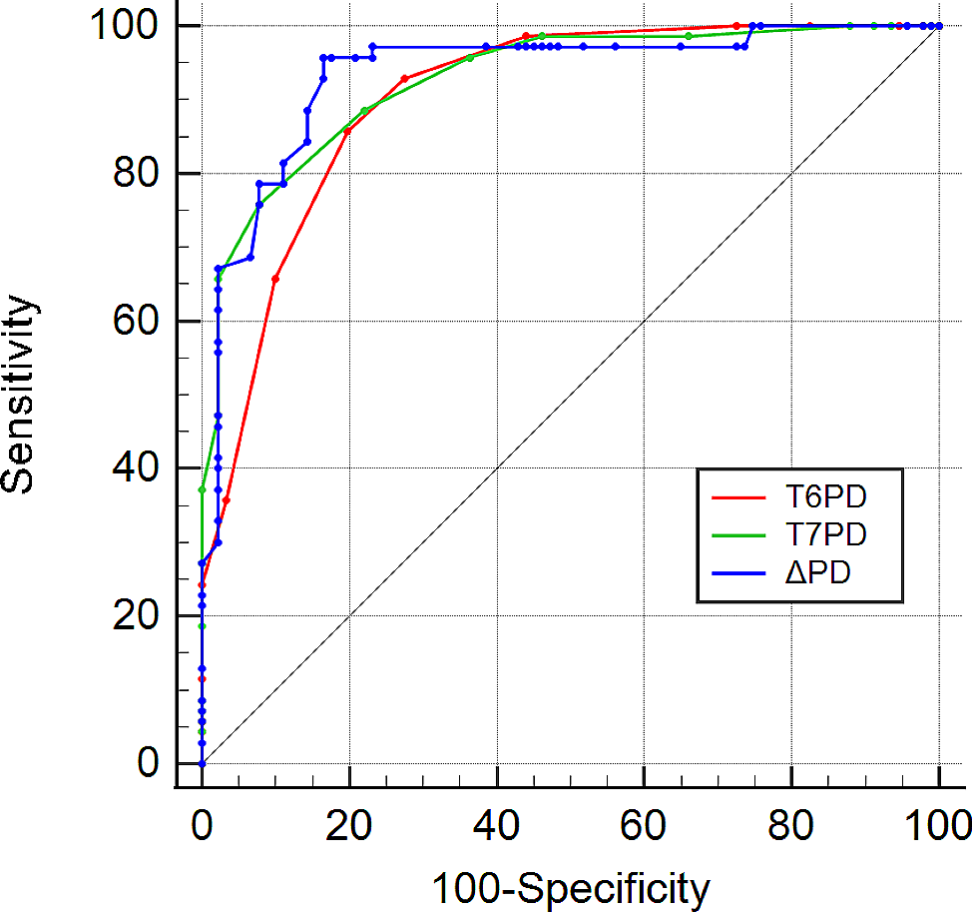
ROC curve of PD and ΔPD predicting VRS ≥ 1. T_6_: at the end of surgery, T_7_: shortly after extubation

The ROC curve analysis showed that the threshold values of T_6_PD, T_7_PD, and T_6_ΔPD to predict pain after awakening were 2.05 mm, 2.25 mm, 20.53%, and sensitivity were 0.86, 0.76, 0.79, and Specificity were 0.80, 0.92, 0.92, respectively. In addition, AUC of all exceeded 0.9 indicates the accuracy prediction(AUC: 0.90, 95%CI: 0.85–0.94; 0.92, 95%CI: 0.88–0.96; 0.93, 95%CI: 0.89–0.97) (Fig. 3).

## Discussion

To exclude the influence of age, gender, BMI, surgical type, etc. on PD, we compared the demographic data of two groups of patients and discovered comparability of data between the pain group and the painless group. Compared with PD2, there was an increase 10s after surgical incision, and the increase was more significant in the pain group, indicating that the pupil is not only sensitive to electrical stimulation but also sensitive to surgical skin cutting stimulation. In addition, patients in the pain group had a larger PD indicating greater sensitivity to pain stimulation. The results of the present study suggested that patients in the pain group had significantly larger PD from the start of skin incision to shortly after extubation, indicating that PD was related to intraoperative nociception, and the accumulation of intraoperative nociception led to pain after awakening. When PD or ΔPD exceeded 2.05 mm or 20.53% at the end of surgery, patients may have postoperative pain, providing a reliable basis for early titration analgesia after surgery.

Regional anesthesia has advantages in orthopedic surgery, but in our study, in order to provide better sedation, analgesia, and muscle relaxation, we included patients with joint replacement under general anesthesia. Conventional pain assessment methods include visual analogue scale (VAS), numerical rating scale (NRS), and verbal rating scale (VRS), while VRS is mainly favored by patients as the easiest method to understand, as well as being more feasible to assess pain [10]. Thus, VRS was adopted for pain assessment in this study, which was consistent with Aissou M’s findings [1]. Although various scales are the gold standard for pain assessment, they are difficult to implement immediately after general anesthesia. Residual anesthetics mainly prevent patients from expressing pain clearly and objectively, distinguishing pain from discomfort within minutes after surgery [10]. Accurate pain assessment is a prerequisite for precise pain management. The PD measurement affected by noxious stimulation and analgesia may provide a valuable index for predicting pain after awakening.

TK500A is a portable electronic pupillary measurement instrument. After capturing the pupillary image, the sensor can directly calculate PD and PLRV. In our study, the ambient light in the operating room was stable and slightly influenced monitoring. After incision, patients in the pain group had a larger PD on the whole. The reason may be that the noxious stimulation excites the inhibitory neurons of the brainstem and strengthens the inhibition of the Edinger-Westphal nucleus. The passive pupillary enlargement causes the PD to become larger [11–15]. At the same time, the degree of PRD is positively correlated with the intensity of noxious stimulation [5]. The application of opioids inhibits pupillary dilation after noxious stimulation by blocking the nerve conduction of the brainstem inhibitory nerves to the Edinger-Westphal nucleus [8]. Therefore, theoretically, PD dynamically reflects the balance between noxious stimulation and analgesia in patients. Enlargement of PD increases the risk of postoperative pain.

The use of opioids may not only make the pupillary constrict but also inhibit the PRD after noxious stimulation. In awake patients, it excites the Edinger-Westphal nucleus directly, leading to pupillary contraction. When the conscious patient was titrated with morphine (0.1 mg kg^-1^), the pupil would shrink within 1 mm for about 4 min [16]. However, under general anesthesia and early awakening, opioids mainly inhibit PRD after noxious stimulation, rather than PD [1]. If the anesthetized patient is not stimulated during surgery, fentanyl can only inhibit PRD after incision and has no significant effect on the baseline PD [4]. It has been proved that the increase in remifentanil concentration is associated with the decrease of PRD during propofol anesthesia in healthy individuals [7]. Therefore, opioids have a direct influence on conscious patients’ PD, however, under general anesthesia, they mainly inhibit the passive pupillary dilation after noxious stimulation, which may be attributed to the indirect excitatory effect of opioids on the Edinger-Westphal nucleus after anesthesia, in which they relieve the inhibitory effect of the noxious stimulation on the Edinger-Westphal nucleus.

General anesthesia even includes drug residues in the early recovery period, and the pupillary oscillation is significantly inhibited by propofol and opioids [17, 18], which is beneficial for accurate measurement of PD and makes it possible to carry out pupillary research. The correlation analysis between the patient’s pain score and PD showed that there was no correlation between preoperative or postoperative pain and PD. The correlation was only found after skin incision, and the correlation coefficient increased as the procedure progressed. Possible reasons are that an irregular pupillary oscillation affects measurement in awake patients and PD assessment of pain perception depends on the presence of noxious stimulation or changes in the patient’s perception [4, 18]. Aissou M [1] first assessed the patient’s pain level with pupillary indicators in the recovery room. No correlation between PD and VRS score was found at 10 min after tracheal tube removal. PD was significantly correlated with the patient’s VRS score only when incision edge was compressed. Different from Aissou M’s findings, the periods used in our study were early and did not need painful stimulation. Pupillary changes reflect the degree of analgesia and sedation, while smooth muscle movements are not affected by neuromuscular blocking agents [18–20]. Thus, BIS during general anaesthesia should be controlled between 40 and 60. After excluding cardiovascular active drugs, anticholinesterase, and anticholinergic drugs, the PD reflects the intensity of noxious stimulation and the balance of opioid analgesia [21–25], justifying why PD can accurately predict the degree of pain after surgery. However, despite the increase in BIS in patients after extubation, the prediction is still accurate at T_7_, which may be related to stimulation of extubation or the change in patients’ nociception.

It is noteworthy that PLRV is the ratio of the difference between the diameter before light on and the minimum diameter after contraction to the time. There was no significant difference in PLRV between the pain group and the painless group in this study, indicating that noxious stimulation and opioids did not affect the nerve conduction velocity through the optic nerve. The light reflex pathway did not innervate the Edinger-Westphal nucleus through central inhibitory neurons, while directly connected with the Edinger-Westphal nucleus, which indirectly validated Larson M’s findings [8, 22].

In the present study, although patients in the pain group were older and used more remifentanil after surgery, no significant difference was found. The intraoperative dose of opioids is not merely an influential factor of postoperative pain and may be associated with individual differences. Thus, the dose of opioids cannot be used to predict postoperative pain, whereas the individual’s sensitivity to opioids can be specifically expressed in pupillary changes [26–30].

In conclusion, PD is a valuable index for evaluating pain perception after awakening in patients undergoing general anesthesia, which can provide early feedback for the patient’s degree of nociception, as well as being a reference for precise pain management after surgery.

## Data Availability

The datasets generated and/or analyzed during the current study are not publicly available due [REASON WHY DATA ARE NOT PUBLIC] but are available from the corresponding author on reasonable request.
